# Are census data accurate for estimating coverage of a lymphatic filariasis MDA campaign? Results of a survey in Sierra Leone

**DOI:** 10.1371/journal.pone.0224422

**Published:** 2019-12-19

**Authors:** Wogba Kamara, Kathryn L. Zoerhoff, Emily H. Toubali, Mary H. Hodges, Donal Bisanzio, Dhuly Chowdhury, Mustapha Sonnie, Edward Magbity, Mohamed Samai, Abdulai Conteh, Florence Macarthy, Margaret Baker, Joseph B. Koroma

**Affiliations:** 1 Statistics Sierra Leone, Circular Road, Tower Hill, Freetown, Sierra Leone; 2 RTI International, Washington, DC, United States of America; 3 Helen Keller International, New York, NY, United States of America; 4 Helen Keller International, Freetown, Sierra Leone; 5 RTI International, Rockville, MD, United States of America; 6 Ministry of Health and Sanitation, Freetown, Sierra Leone; 7 University of Sierra Leone, Freetown, Sierra Leone; 8 Neglected Tropical Disease Control Program, New England, Freetown, Sierra Leone; 9 FHI360, Accra, Ghana; Syracuse University, UNITED STATES

## Abstract

**Background:**

Preventive chemotherapy was administered to 3.2 million Sierra Leoneans in 13 health districts for lymphatic filariasis, onchocerciasis, and soil transmitted helminthes from October 2008 to February 2009. This paper aims to report the findings of a coverage survey conducted in 2009, compare the coverage survey findings with two reported rates for lymphatic filariasis coverage obtained using pre-mass drug administration (MDA) registration and national census projections, and use the comparison to understand the best source of population estimates in calculating coverage for NTD programming in Sierra Leone.

**Methodology/Principal findings:**

Community drug distributors (CDDs) conducted a pre- MDA registration of the population. Two coverage rates for MDA for lymphatic filariasis were subsequently calculated using the reported number treated divided by the total population from: 1) the pre-MDA register and 2) national census projections. A survey was conducted to validate reported coverage data. 11,602 persons participated (response rate of 76.8%). Overall, reported coverage data aggregated to the national level were not significantly different from surveyed coverage (z-test >0.05). However, estimates based on pre-MDA registration have higher agreement with surveyed coverage (mean Kendall’s W = 0.68) than coverage calculated with census data (mean Kendall’s = 0.59), especially in districts with known large-scale migration, except in a highly urban district where it was more challenging to conduct a pre-MDA registration appropriately. There was no significant difference between coverage among males versus females when the analyses were performed excluding those women who were pregnant at the time of MDA. The surveyed coverage estimate was near or below the minimum 65% epidemiological coverage target for lymphatic filariasis MDA in all districts.

**Conclusion/Significance:**

These results from Sierra Leone illustrate the importance of choosing the right denominator for calculating treatment coverage for NTD programs. While routinely reported coverage results using national census data are often good enough for programmatic decision making, census projections can quickly become outdated where there is substantial migration, e.g. due to the impact of civil war, with changing economic opportunities, in urban settings, and where there are large migratory populations. In districts where this is known to be the case, well implemented pre-MDA registration can provide better population estimates. Pre-MDA registration should, however, be implemented correctly to reduce the risk of missing pockets of the population, especially in urban settings.

## Introduction

Neglected Tropical Diseases (NTDs) such as lymphatic filariasis (LF), onchocerciasis, schistosomiasis, soil-transmitted helminthes (STH), and trachoma burden the lives of 2.7 billion people, mostly in Africa, Asia, and the Americas [1, 2]. Worldwide, the chronic suffering, disfigurement, and disability imparted by NTDs compete with those caused by HIV/AIDS, tuberculosis, and malaria, resulting in up to 57 million disability-adjusted life years lost annually, impacting the socioeconomic status of households, communities, and nations [2, 3]. A small country in West Africa, Sierra Leone’s population in 2008 was estimated to be 5.5 million, with the entire population at-risk of LF and STH, 2.7 million at risk of onchocerciasis, and 2.1 million at risk of schistosomiasis [unpublished data, Sierra Leone Ministry of Health] [4]. This high burden of NTDs in a country ranked among the last in the Human Development Index threatens its potential to achieve significant progress in health, education, and economic development [5].

Given the geographic overlap of onchocerciasis, LF, and STH in Sierra Leone, integrated mass drug administration (MDA) is conducted annually. Ivermectin (IVM), donated by Merck & Co. Inc. (Whitehouse Station, New Jersey, USA), used to treat onchocerciasis, is combined with albendazole (ALB), donated by GlaxoSmithKline (Middlesex, United Kingdom), to treat LF, simultaneously serving as deworming agents for STH [6–8].

In Sierra Leone, drugs are delivered annually to the community by community drug distributors (CDDs). The CDDs are trained to conduct a pre-MDA registration to capture the individuals in their catchment area, mobilize those individuals to participate in the MDA, distribute the drugs, document the treatments on data forms, and submit those forms to the Peripheral Health Unit (PHU) staff after the MDA. Data from each PHU are then compiled at district-level and sent to the national NTD control program, which uses the data to calculate MDA treatment coverage. These coverage data are used at various levels within the country to monitor performance of the MDA to reach the necessary proportions of at-risk and targeted individuals in order to decrease transmission. Additionally, these coverage data are reported to WHO and donors, as a measure of program quality as well as to facilitate monitoring progress towards national, regional, and global goals.

WHO guidance for monitoring drug coverage in most national NTD programs is that the denominator is best estimated from national census projections. WHO recommends that in cases where the national census may not be updated, is considered inaccurate, or may exclude certain populations, such as nomadic populations, countries may choose an alternative data source, such as a pre-MDA census, to more accurately represent the implementation unit [9, 10]. For onchocerciasis, APOC’s recommendation was that pre-MDA registration of the population be conducted for use as the source of the denominator for the calculation of coverage when community-directed treatment was conducted [11].

The decade-long civil war from 1991–2002 catalyzed the mass movement of populations from Sierra Leone’s rural areas to the Western Area around the capital, resulting in almost one fifth of the country’s population to reside in Freetown. Population migration back to the rural areas has occurred in recent years due to an increased sense of security in the villages as well as relocation in search of economic opportunities. These shifts in population were not captured in the 2004 national census; however, they were anecdotally understood to be better captured in the pre-MDA register conducted annually by the CDDs. Therefore, the national NTD program faced the question of which population estimate was more accurate, and therefore should be used for calculating and monitoring coverage at the district level. Other public health programs around the world have also cited challenges with obtaining accurate population estimates for use in planning and monitoring coverage [12–17].

In this paper, we report findings of a survey conducted post-MDA to estimate epidemiological coverage, supported by the United States Agency for International Development (USAID). The aim of the survey was to assess whether in Sierra Leone the surveyed coverage rates are closer to reported coverage rates using projected national census data as the denominator or to those using the NTD program’s pre-MDA register data as the denominator. We also report data on surveyed coverage by age and gender.

## Methods

### Ethical considerations

This study was conducted according to the principles of the Helsinki Declaration and approved by the Ethics Committee of the Ministry of Health and Sanitation (MOHS), Sierra Leone. Verbal informed consent to administer the questionnaire and analyze the data was obtained from all village chiefs, household heads, and all adults and children interviewed; parents provided verbal informed consent for young children (with parental consent, respondents <18 years of age were typically interviewed in front of their parents, and parents responded for young children). Verbal informed consent was obtained due to the low literacy rates across the country [18]. Names were not collected; each person interviewed was given a unique identification number, which was entered into the database.

### Definitions of MDA coverage

In this paper we refer to epidemiological coverage for MDA as the total number of individuals treated through MDA divided by the total number of individuals living in an endemic area (i.e., at risk for the disease, regardless of whether they are eligible for treatment), in line with WHO’s definition of epidemiological coverage [10]. The decision to use the total population as the denominator, rather than the eligible population targeted, is aligned with the WHO-defined minimum coverage threshold that at least 65% of the total population should be treated during each MDA for LF [10].

$$Epidemiological\;coverage=\frac{\#individuals\;who\;received\;MDA\;treatment}{\#individuals\;living\;in\;an\;NTD‐endemic\;area}\;*100$$

Epidemiological coverage can be measured through routinely reported data (reported coverage) or through periodic coverage evaluation surveys (surveyed coverage). The numerator for reported coverage is the number treated as those reported by CDDs during the campaign. This is divided by estimates of the total population living in an NTD-endemic area. In this paper, we indicate reported coverage separately for when national census projection data are used for the denominator and for when NTD program pre-MDA register data are used. The numerator for surveyed coverage is the total number persons who indicated during the survey to have swallowed the drugs; the denominator for surveyed coverage is the total number persons interviewed in an NTD-endemic area.

### Mass drug administration and reported coverage estimates

During MDA from October 2008 to February 2009, 13 of the 14 Health Districts endemic for LF were targeted for treatment with IVM and ALB [unpublished data, Sierra Leone Ministry of Health]. Among these 13 districts, STH was widespread and onchocerciasis was also moderately/highly endemic in 8,451 villages. CDDs exclusively used the house-to-house strategy in eight districts which had only small non-rural populations. The other 5 districts used this house-to-house strategy as well as community-based distribution in the headquarter towns with distribution taking place in markets, mosques and other busy non-rural locations.

#### Determining the reported numerator

During the MDA, CDDs recorded on treatment registers to whom they administered drugs, in addition to other characteristics of each of the household members such as age, sex, and select eligibility status. Once their work was completed, the CDDs reported the number of persons treated to their PHU supervisors, who compiled these reports and forwarded them to their district supervisors. All reports were collected by the national NTD program within two weeks of completion of MDA.

##### Estimating the denominator using national census projections

The national census was performed in 2004 by the national statistics office [4]. The population in 2008 was projected in each district using an annual median growth rate of 2.5%. Estimates of ineligible groups (e.g. pregnant women, children <90 cm in height, etc.) were not excluded from the census-based denominator.

##### Estimating the denominator using program pre-MDA register data

In August 2008, PHU staff trained CDDs to take a pre-MDA register of each household member regardless of MDA exclusion criteria. The pre-MDA register process consisted of the CDD traveling to their designated villages in the weeks preceding MDA to document the name, age, and sex of all inhabitants of each household in a register. On average, two CDDs worked together to cover a catchment area of approximately 500 persons consisting of two to three villages, depending on the local context. The information gathered for the pre-MDA register was then used to forecast drug needs.

#### Independent coverage survey sampling

In April 2009, a post-MDA coverage evaluation survey was conducted in Sierra Leone to validate the reported epidemiological coverage values. As the MDA epidemiological coverage was unknown during the preliminary design of the survey, it was assumed that within a 95% confidence level, the coverage was 50% of the total population with a design effect of 5 and precision of 5%, based on recommendations from previous NTD drug coverage surveys [19]. The total population was the general household population of the 13 health districts targeted with IVM and ALB distribution during the 2008 MDA campaign [4]. Using power calculations, we estimated the sample size to be 13,200, taking into account a non-response rate of 10%. The total sample size was divided by the average household size of six persons to determine that 2,200 households should be surveyed across the 13 districts [18].

A sampling frame of primary sampling units was obtained from a complete list of enumeration areas (EA) based on the national census population projections. Each EA contained the following: target population projections; socioeconomic and demographic information; officially recognized geographical, administrative classifications; and cartographic tools with identifiable boundaries. The sample was randomly selected using three-stage cluster sampling methodology. In the first stage, a total of 220 EAs were selected from 8,322 EAs, using probability proportionate to size (PPS) to allocate clusters to the 13 targeted districts in a self-weighting sample [20]. In the second stage, when an EA constituted two or more villages, a list of those villages was made and one randomly selected; however, most EAs consisted of only one village. In the third stage, a list of households defined as those who live under the same roof, eat daily from the same cooking pot, and recognize one person as the head-of-household, served as the sampling frame for ten randomly chosen households per EA. Data from all members in each selected household were recorded for the interview. While this assessment was designed as a national survey, there was sufficient power to calculate intervals at the district level as well.

#### Interviewers and questionnaire

Four teams comprising a supervisor from Statistics Sierra Leone and four interviewers were recruited. The interviewers were college graduates and/or health care workers with experience in data collection. In order to standardize the fieldwork, a one-day training session was conducted to prepare the interviewers for this survey. During the training, prompting for a response to questions by naming a significant village event that may have coincided with MDA such as an election or a wedding, demonstration of the dose pole, naming the village’s CDD or showing the tablets was recommended, in order to minimize recall bias.

Once a household was selected, teams first interviewed the head-of-household and then household members; if children were unwilling or unable to respond for themselves, a parent or caretaker in the household was asked to answer the questions on their behalf. If an adult household member was absent during the time of the survey, his/her age and sex were recorded on the survey form, and it was indicated on the survey form that they were not present at the time of the survey. A closed-ended questionnaire was orally administered to all survey participants, including the question whether or not the respondent swallowed IVM and/or ALB, and if one or both of the drugs was not taken, then the reason why. Interviews were conducted in Mende, Themne, Limba, or Krio, based on the local language spoken in the household.

### Statistical analysis

The data were hand recorded on questionnaires, entered into EpiInfo 3.5.1 (CDC, Atlanta, GA, USA) by staff at Statistics Sierra Leone, validated by double checking the database against hard copies, and analyzed using SAS 9.2 (SAS Institute, Cary, North Carolina, USA), SUDAAN 10 (RTI International, Research Triangle Park, North Carolina, USA), and R language (version: 3.5.1, [21]). The design weights were calculated using the overall probability of selection for individuals; that is, the product of the probabilities of selection from the three stages of sample selection: districts, villages and households. The design weight is the inverse of the probability of selection for people in sampled households and zero for non-sampled households. Non-response adjustments were computed to correct the sample weights. The “WTADJUST” procedure in SUDAAN was used for non-response using a generalized exponential model. All data were analyzed and presented in aggregate. Generalized linear mixed model (GLMM) were used to perform a logistic regression to investigate relationship among age, sex, and districts on being treated with IVM or ALB during MDA. Modeling was performed using structured additive regression models (STAR) performed with BayesX software [22]. The STAR model allows the inclusions of non-linear variables such as spatial random effect. The STAR models used for this study included districts as spatial effect as Markov random fields [23]. This model variable was used to account for the spatial relationship among the districts. A post-hoc Tukey’s test [24] was applied to the logistic regressions to investigate coverage differences between female and male coverage at district levels per each drug. Design effects were calculated to measure the impact of the study design features: stratification and clustering; unequal weights; and over or under sampling.

Surveyed coverage was disaggregated by district, sex, age and the reasons given for not taking drugs were recorded; these values were reported separately for IVM and ALB, as it is possible that a person may have taken one drug but refused the other, or the other had not been available.

To address the aim of determining which population estimate is more accurate, the surveyed coverage rates were then compared with coverage estimates using the CDD treatment register data as the numerator, and the two denominator options: census projection and pre-MDA registration. This was done for any districts where the surveyed coverage precision was sufficient, as defined by the 95% confidence intervals spanning less than +/- 20 percentage points; any range greater than that was determined to be insufficient for making conclusions. In comparing the reported and surveyed coverage rates, we defined "discrepancy" as the number of percentage points the reported coverage estimate was away from the surveyed coverage point estimate. Comparison among coverage calculated with national census, pre-MDA registration, and survey methods was done using z-tests [25]. Kendall’s W was calculated to calculate which among the two reported coverage methods had the highest concordance with the surveyed estimates. The z-test and Kendall’s W were performed using applying boot-strapping in order to account for the uncertainty of survey estimations. All tests and models included the sample weight to account for the sampling design.

## Results

### Survey sample size and respondents

A total of 2,200 households were visited, and age and sex data collected for 14,595 individuals including present and absent household members. In a number of districts, a large number of individuals were absent at the time of the survey. Due to limited resources, these households were not revisited for follow-up. Of the 14,595 household members (males: 45.2%, females: 54.8%), 11,206 agreed to participate in the survey yielding a response rate of 76.8%. Of these respondents, 11.8% were pre-school aged children (<5 years), 29.6% were school aged children (5–14 years), 26.1% were young adults (15–29 years old) and 32.5% were older adults (≥30 years). The design effect was 14.79 for ALB and 14.04 for IVM.

### Comparison of coverage rates

#### Reported coverage–using census vs pre-MDA registration as denominator

**Fig 1:**
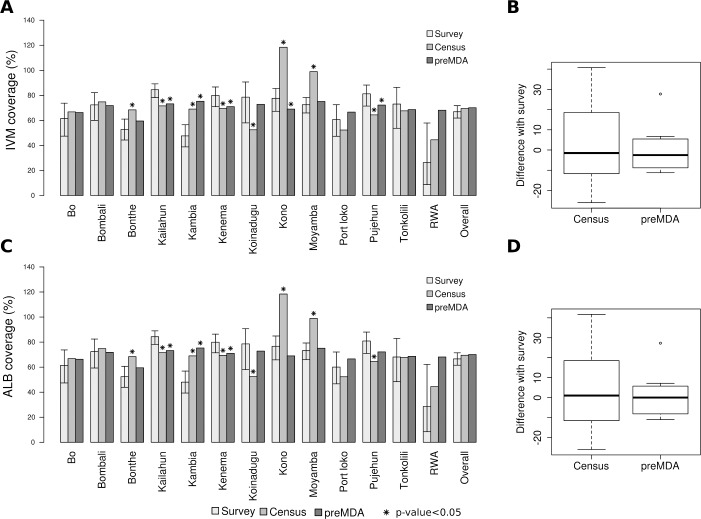
Coverage calculated using reported treatment coverage–with both pre-MDA registration and census denominators–and post MDA coverage survey. The panels show IVM (A) and ALB (C) coverage calculated using reported coverage (with either census or pre-MDA registration as the denominator) and survey methods. A significantly statistical difference between surveyed and reported coverage rates is shown with an *. Panels B and D use boxplots to show the distribution of district level differences between surveyed coverage and reported coverage rates.

The overall reported coverage obtained using the pre-MDA register was 70.1%, as shown in Table 1 and Fig 1. At the district level, the highest coverage reported using the pre-MDA register was in Kambia at 75.3% and Moyamba at 75.1%, and the lowest in Bonthe at 59.5% and Port Loko at 66.6%. Reported coverage obtained using the national census projections was very similar to the pre-MDA registration value when aggregated to the national level, at 69.5% (Table 1 and Fig 1). However, there were differences at the district level; the highest coverage calculated with the national census projections was recorded in Kono at 118.4% and Moyamba at 98.9%, while the lowest was in the Rural WA (RWA) at 44.5%, Port Loko at 52.3%, and Koinadugu at 52.5% (Fig 1).

**Table 1:** MDA coverage calculated using survey data, census population, and pre-MDA population and their comparison.

	Survey (95% CI)	Reported–Census denominator	Reported–pre-MDA registration	Difference Survey and Census in percentage points: point estimate (95% CI)	Difference Survey and pre-MDA in percentage points: point estimate (95% CI)
IVM coverage	66.9% (61.8, 71.7)	69.5%	70.1%	-2.6 (-7.7, 2.2)	-3.2 (-8.3, 1.6)
ALB coverage	66.6% (61.5, 71.4)	69.5%	70.1%	-2.9 (-8.1, 1.9)	-3.5 (-8.6, 1.3)
IVM Kendall’s W	-	0.56	0.64	-	-
ALB Kendall’s W	-	0.59	0.68	-	-

The table shows the difference and agreement among the methods (Kendall’s W).

The five districts where there were larger differences observed (≥10 percentage points) between the reported values using the national census projections and those using pre-MDA registration were Kono, Moyamba, Koinadugu, Port Loko, and RWA. In Kono and Moyamba, the coverage values using the national census estimates were substantially higher than estimates using the pre-MDA registration. (In Kono, pre-registration population estimates were 466,223 versus 271,733 using national census projections–a difference of 194,490 persons (a 53% difference); in Moyamba, pre-registration population estimates were 309,436 versus 234,963 using the national census projections, a difference of 74,473 persons, or 27%., Fig 1). In Koinadugu, Port Loko and RWA, the registration population estimates were substantially lower than national census estimates, resulting in much higher reported coverage using the pre-MDA registration population estimates (Fig 1). Pre-MDA registration determined a population value of 207,995 in Koinadugu, compared to 288,672 using the national census projections, which is a 32% difference. The population values differed by 102,570, or 24%, in Port Loko. In RWA, there was a 42% difference, with the pre-MDA registration indicating a population of 151,146 while the national census projections showed 231,294 (Fig 1).

### Surveyed coverage

Overall, surveyed coverage with IVM was 66.9% (95% CI: 61.8%, 71.7%) and with ALB was 66.6% (61.5, 71.4), see Table 1. At the district level, the highest surveyed coverage was in Kailahun at 84.4% (95% CI: 78.1%, 89.2%) for both drugs; the lowest was in RWA at 26.3% (95% CI: 8.5–57.9%) for IVM and 28.0% (95% CI: 8.4–62.2%) for ALB (Fig 1).

Similar *national* level coverage rates masked significant *district* level differences in coverage (Table 1, Fig 1 and S1 Table in SI). While the survey method reported national level coverage as only 3 percentage points higher than estimates obtained from reported coverage methods (Table 1), there were significant differences in several, but not all, districts (Fig 1). Of the 12 districts where the coverage survey had sufficient precision (i.e., all but RWA), the surveyed coverage results validated reported coverage using pre-MDA registers in 10 districts and validated reported coverage using national census projections in another 4 districts (Fig 1 and S1 Table in supporting information [SI]). In these districts, surveyed coverage rates were less than 10 percentage points different from reported coverage estimates (RWA is excluded from this analysis due to imprecision of the surveyed coverage estimate). Overall, coverage estimated using census denominators differed more from surveyed coverage than those estimated using pre-MDA registration denominators (Fig 1). The estimates based on pre-MDA registration have higher agreement with surveyed coverage (mean Kendall’s = 0.64 for IVM and Kendall’s = 0.68 for ALB) than coverage calculated with census data (mean Kendall’s = 0.56 for IVM and Kendall’s = 0.59 for ALB).

**Fig 2:**
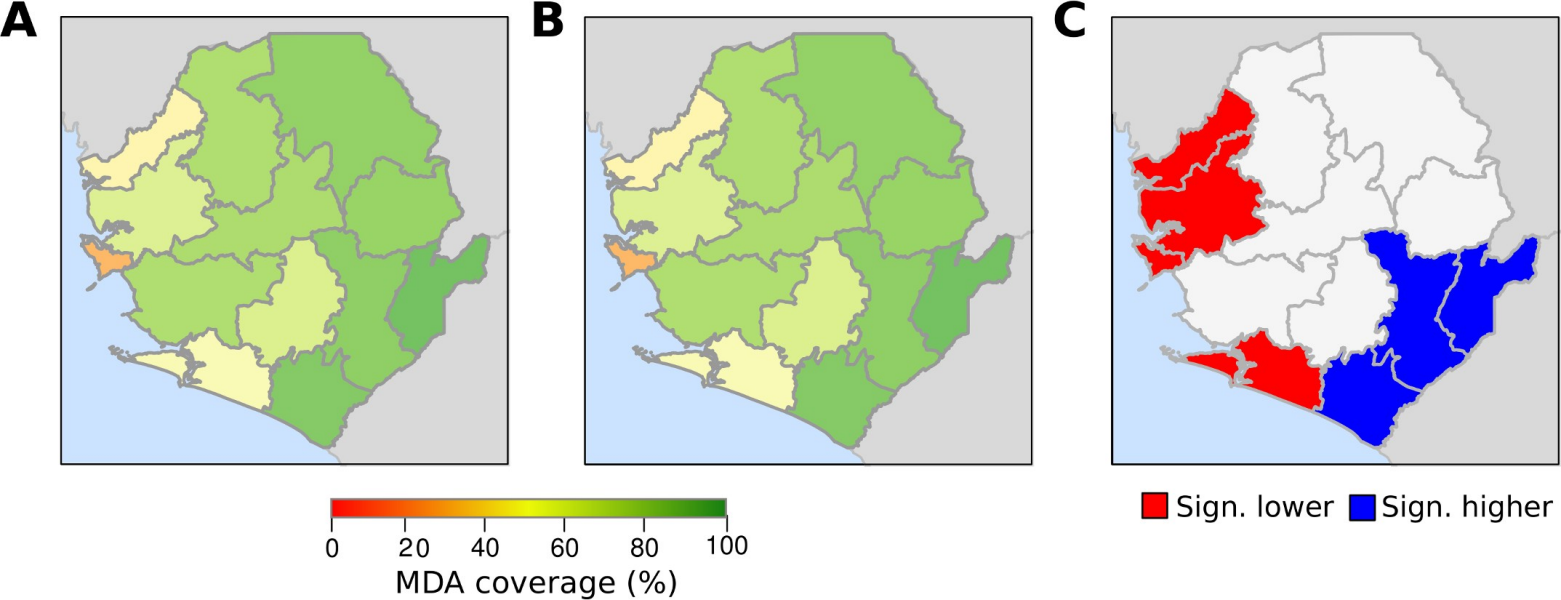
Survey coverage of IVM and ALB at district level and districts with statistically high and low surveyed coverage. The image shows surveyed coverage calculated for IVM (Panel A) and for ALB (Panel B). The figure also includes a map (Panel C) showing significant clusters of districts where people had significant lower and higher probability to take MDA drugs (IVM and ALB, one map is shown because drug models had same results) compared with the rest of districts. This map is the result of the random spatial effect variable of the logistic regression.

The spatial random effect of the logistic regression identified districts where people had statistically higher (3 districts at the border with Liberia) and lower (4 districts in the western part of the country) probability to take drugs administrated during MDA campaign compared with the rest of country’s districts (Fig 2). The spatial random effect of IVM and ALB gave identical results.

#### Survey coverage by age group and sex

Coverage was significantly lower in young adults aged 15–29 years for both IVM and ALB, 64.2% (95% CI: 59.3–68.8) and 63.6% (95% CI: 58.6–68.2), respectively, compared to adults ≥30 years and school aged children (5–14 years) for IVM and ALB, (p<0.05, Table 2). Young adults had 30% less probability to be reached by MDA compared with children below 15 years of age. The ≥30 years age group had higher coverage compared with the other age groups (Table 3). Overall 1.1% (n = 14) of children under five years of age at the time of MDA took IVM (age was documented at the time of the survey).

**Table 2:** Surveyed coverage % (95% confidence interval) by age group, sex at country level.

Age group	% IVM	% ALB
5<15 (Ref. age group)	74.5 (70.3–78.3)	73.6 (69.3–77.6)
15<30	64.2 (59.3–68.8)**	63.6 (58.6–68.2)**
≥30	74.7 (70.9–78.2)**	73.5 (69.4–77.3)**
		
**Sex**		
Male (Ref. = Female)	65.0 (61.4–68.4)*	64.0 (60.3–67.6)*
Female	58.6 (54.7–62.4)	58.1 (54.1–62.0)
No pregnant females (Ref. = Male)	63.1 (58.5–69.8)	64.1 (59.2–68.7)

*p<0.05

**p<0.01, p-value from GLMM logistic regressions (model results are showed in Table 3).

**Table 3:** Results from logistic regression GLMM.

	IVM model OR (95% CI)	ALB model OR (95% CI)
Intercept	1.81 (1.15, 2.84)**	1.83 (1.191, 2.82)**
Sex:		
Female (Ref)	1.0	1.0
Male	1.34 (1.22, 1.47)*	1.30 (1.18, 1.43)*
Age groups:		
5<15 years (Ref)	1.0	1.0
15<30 years	0.71 (0.63, 0.80)**	0.70 (0.62, 0.79)**
≥30 years	1.26 (1.12, 1.42)**	1.18 (1.06, 1.33)**
District^a^	*	*

* = p<0.05

** = p<0.01

^a^spatial random effect showed in Fig 2C

Males had significantly higher probability of being treated during the MDA campaign as compared to females (Table 2 and Table 3). However, this difference was not significant when the analyses were performed excluding those women who were pregnant at the time of MDA (GLMM, p = 0.17). Women in the age group 15–29 years were significantly lower than men of same age group only when pregnant women were not excluded from the analyses (Tukey’s test, p>0.05). Although higher coverage among males was found in each district, with the exclusion of Bonthe, this difference was not significant at district level (Tukey’s test, p>0.05) (S2 Table in SI). No significant difference was found comparing coverage of IVM and ALB by sex and age groups (z-test, p>0.05). The difference between the coverage of the two drugs was negligible, with the district level difference equal to approximately 0.5 percentage points.

## Discussion

Gaining a true understanding of treatment coverage is vital to measuring the outcome of MDA; the higher the treatment coverage, the greater chance a program has of elimination of LF [26]. Elimination of LF can be achieved in endemic areas through the distribution of IVM and ALB to at least 65% of the at-risk population annually for five or six years. Because accurate calculations of the coverage of the MDA plays such an important role in monitoring the progress made towards the interruption of disease transmission, and the timing of impact assessments, the World Health Organization recommends that national NTD programs periodically conduct post-MDA coverage surveys [10]. Post-event coverage surveys have also proven to be useful in public health initiatives for malaria, polio, measles, yellow fever, and expanded immunization [12, 16, 27–30], in addition to their use in NTD programs [31–34].

### Comparison of surveyed coverage rates to reported coverage rates

In this paper we report that coverage surveys validated reported coverage data in about half of the 13 districts surveyed for LF treatment coverage, regardless of the source of the denominator. However, in two districts–Kono and Moyamba–coverage estimates using reported coverage rates with national census data were greatly overestimated. These two districts experienced higher-than-projected post-conflict population growth due to the alluvial diamond mining and the re-opening of the rutile mines, respectively, resulting in post-war employment opportunities. Population increases in these districts were more accurately captured by the pre-MDA registers.

On the other hand, pre-MDA registration substantially underestimated the population size in RWA. This district has seen the greatest degree of growth in the post-conflict era due to the settlement of internally-displaced persons in multiple, unplanned, unregulated, and un-enumerated areas, especially in the parts in closest proximity to the capital city of Freetown. The smaller population size estimated by pre-MDA registration was not accepted at the time by consensus of the national NTD program and its partners, who recognized that the data did not reflect what was known about the population in that district. They also recognized that the health infrastructure in this district was overstretched due to the rapid population growth with correspondingly few CDDs. It was in fact determined that many new settlements in RWA (which did not exist as entities in the 2004 census) had been excluded by the CDD pre-MDA registration as they fell outside the recognized catchment area of their PHU. It is also of note that the surveyed coverage confidence intervals for this district are particularly wide, possibly reflecting a high design effect here due to patchy low and high drug coverage in sampled clusters.

These results demonstrate the challenges national programs face in accurately determining population estimates. National NTD programs must therefore make an informed decision to choose the most accurate source available for the denominator, recognizing that there are limitations in the options available. Due to the larger inaccuracies of the national census projections in some of the districts, and the similarity with the pre-MDA register values in the other districts, the national NTD program in Sierra Leone chose to use the population estimates derived from the pre-MDA CDD registration for all of the districts surveyed except RWA. This decision acknowledges both the difficulty that the CDDs would face in accurately capturing all the residents in urban areas during pre-MDA registration, and also the substantial migration that has occurred within post-war Sierra Leone as people return home as well as migrate for economic opportunities which makes the national census projections inaccurate. Other situations where census data is commonly incorrect include districts with large nomadic populations [9].

### Survey limitations

The non-response rate of 23.2% was higher than the anticipated 10%, which may have introduced bias. Specific measures were not consistently in place to ensure that interviewers prompted the respondent about the day MDA occurred, even though this was recommended during the training. The use of an English questionnaire orally translated by the interviewer rather than pre-tested translations into the local languages could also have introduced response bias. In the village setting, it was difficult to isolate household members during the interviews, thus the answers given by one member could have been overheard and influenced the answers from others, particularly when children overheard the responses of adults.

This survey did not collect data on side effects that could have resulted from consumption of IVM and ALB in previous round(s) of MDA and could have thus, perhaps, impacted compliance in the 2008 round [35, 36]. Additionally, the survey was conducted in villages where multiple health campaigns had taken place in rapid succession through the PHUs, often implemented by the same CDDs. This could have led to confusion by respondents when asked questions about one specific campaign: MDA for LF, onchocerciasis, and STH. The survey did not use the pre-MDA register data as a basis for the sampling frame for choosing survey locations using PPS, but rather used data from the national census population projections.

Finally, this study did not included other information sources of country population freely available such WorldPop [37] and LandScan [38]. These projects provide fine resolution country population data obtained by disaggregating projected census data by using remote sensing data (e.g., nightlight, landuse). Thus, we believe that adding population estimates at the district level based on Worldpop and LandScan images would result very similar to our method based on census data.

## Conclusions

WHO guidance recommends that national census data be routinely used as the denominator for estimating treatment coverage but that “if the official census is considered inaccurate, the [implementation unit] IU should judge which source most accurately reflects its total population” [10] (page 13). These results from Sierra Leone illustrate both the importance of the nuance in this guidance and the importance of decision making about the use of the most contextually appropriate data sources by national program managers. In many cases, routinely reported coverage results using national census data are good enough for programmatic decision-making. However, in some cases, census projections can quickly become outdated where there is substantial migration e.g. due to the impact of civil war, with changing economic opportunities, in urban settings, and/or where there are large migratory populations. Given the importance that routine coverage data play in monitoring and evaluation of NTDs, in districts where this is known to be the case, well implemented pre-MDA registration can provide better population estimates. Pre-MDA registration should, however, be implemented correctly to reduce the risk of missing pockets of the population, especially in urban settings.

## Supporting information

S1 Table Comparison of coverage rates by district using pre-MDA register populations, national census population projections, and the coverage survey. (DOCX)

S2 Table Surveyed coverage % (95% confidence interval) by sex per district. (DOCX)
