# Supplementary material for: Are census data accurate for estimating coverage of a lymphatic filariasis MDA campaign? Results of a survey in Sierra Leone
Source: PLoS One. 2019 Dec 19;14(12):e0224422. doi: 10.1371/journal.pone.0224422 (PMC6922463; doi:10.1371/journal.pone.0224422)
Supplement: S1 Table — (DOCX) [file pone.0224422.s001.docx]

**S1 Table.** **Comparison of coverage rates by district using pre-MDA register populations, national census population projections, and the coverage survey.**

N indicates the number of persons at risk for LF, STH, and possibly onchocerciasis using population information from the 2008 pre-MDA register or 2008 median variance projections of the 2004 national census, as indicated in the column heading

n indicates the total number of persons sampled during the survey who responded to the question. Each person therefore represents a person at risk for LF, STH, and possibly onchocerciasis

| **District** | **Drug** | **A**  **Reported coverage % (N) using pre-MDA register** | **B**  **Reported coverage % (N) using national census** | **C**  **Surveyed coverage %, 95% CI (n)**  **from coverage survey** |
| --- | --- | --- | --- | --- |
| Bo | IVM | 66.3 (574,053) | 66.8 (570,171) | 61.4, 47.4-73.7 (871) |
|  | ALB | 66.3 (574,053) | 66.8 (570,171) | 61.4, 47.4-73.7 (871) |
| Bombali | IVM | 71.8 (440,932) | 74.8 (423,625) | 72.3, 59.9-82.1 (1,048) |
|  | ALB | 71.8 (440,932) | 74.8 (423,625) | 72.3, 59.2-82.4 (1,048) |
| Bonthe | IVM | 59.5 (166,140) | 68.4 (144,501) | 52.7, 44.3-61.1 (416) |
|  | ALB | 59.5 (166,140) | 68.4 (144,501) | 52.3, 43.8-60.7 (416) |
| Kailahun | IVM | 73.2 (392,819) | 71.6 (401,389) | 84.4, 78.1-89.2 (995) |
|  | ALB | 73.2 (392,819) | 71.6 (401,389) | 84.2, 78.1-88.9 (995) |
| Kambia | IVM | 75.3 (269,673) | 69.0 (294,049) | 47.6, 38.8-56.5 (734) |
|  | ALB | 75.3 (269,673) | 69.0 (294,049) | 48.1, 39.3-56.9 (734) |
| Kenema | IVM | 71.0 (551,797) | 69.4 (564,936) | 79.9, 70.9-86.7 (1,216) |
|  | ALB | 71.0 (551,797) | 69.4 (564,936) | 79.9, 71.4-86.3 (1,216) |
| Koinadugu | IVM | 72.8 (207,995) | 52.5 (288,672) | 78.6, 58.1-90.7 (742) |
|  | ALB | 72.8 (207,995) | 52.5 (288,672) | 78.6, 58.1-90.7 (742) |
| Kono | IVM | 69.0 (466,223) | 118.4 (271,733) | 77.7, 67.3-85.5 (833) |
|  | ALB | 69.0 (466,223) | 118.4 (271,733) | 76.7, 65.8-84.9 (833) |
| Moyamba | IVM | 75.1 (309,436) | 98.9 (234,963) | 72.5, 66.1-78.2 (676) |
|  | ALB | 75.1 (309,436) | 98.9 (234,963) | 59.1, 52.8-65.0 (676) |
| Port Loko | IVM | 66.6 (376,212) | 52.3 (478,782) | 60.6, 47.3-72.5 (1,153) |
|  | ALB | 66.6 (376,212) | 52.3 (478,782) | 60.1, 46.7-72.1 (1,153) |
| Pujehun | IVM | 72.2 (261,509) | 64.4 (293,509) | 81.2, 71.4-88.2 (449) |
|  | ALB | 72.2 (261,509) | 64.4 (293,509) | 80.9, 70.9-88.1 (449) |
| Tonkolili | IVM | 68.6 (368,678) | 67.6 (373,953) | 73.1, 53.7-86.4 (919) |
|  | ALB | 68.6 (368,678) | 67.6 (373,953) | 68.1, 48.4-82.9 (919) |
| RWA | IVM | 68.1 (151,146) | 44.5 (231,294) | 26.3, 8.5-57.9  (559) |
|  | ALB | 68.1 (151,146) | 44.5 (231,294) | 28.0, 8.4-62.2  (559) |
| Overall | IVM | 70.1 (4,536,613) | 69.5 (4,571,577) | 66.9, 61.8 -71.7 (11,200) |
|  | ALB | 70.1 (4,536,613) | 69.5 (4,571,577) | 66.9, 61.8-71.7 (11,200) |
