# Supplementary material for: Are census data accurate for estimating coverage of a lymphatic filariasis MDA campaign? Results of a survey in Sierra Leone
Source: PLoS One. 2019 Dec 19;14(12):e0224422. doi: 10.1371/journal.pone.0224422 (PMC6922463; doi:10.1371/journal.pone.0224422)
Supplement: S2 Table — (DOCX) [file pone.0224422.s002.docx]

**S2 Table. Surveyed coverage % (95% confidence interval) by sex per each district.**

|  |  |  |  |
| --- | --- | --- | --- |
| **Sex** | | **% Male** | **% Female** |
| Bo | IVM | 56.9 (48.3-65.1) | 50.5 (37.7-62.4) |
|  | ALB | 56.4 (47.5-64.9) | 50.1 (37.7-62.4) |
| Bombali | IVM | 72.7 (60.3-82.4) | 73.1 (60.2-83.0) |
|  | ALB | 73.3 (60.8-82.9) | 71.4 (58.0-81.9) |
| Bonthe | IVM | 62.8 (54.0-70.8) | 64.8 (56.4-72.3) |
|  | ALB | 62.8 (54.0-70.8) | 63.9 (55.6-71.5) |
| Kailahun | IVM | 73.6 (68.5-78.1) | 64.8 (57.3-71.7) |
|  | ALB | 73.1 (68.1-77.6) | 65.0 (57.5-71.8) |
| Kambia | IVM | 61.1 (48.3-72.6) | 49.0 (37.4-60.8) |
|  | ALB | 61.7 (48.9-73.0) | 49.3 (37.6-61.2) |
| Kenema | IVM | 68.8 (61.5-75.2) | 63.2 (55.3-70.4) |
|  | ALB | 68.2 (61.4-74.2) | 63.6 (56.1-70.6) |
| Koinadugu | IVM | 79.4 (58.4-91.4) | 77.1 (56.6-89.6) |
|  | ALB | 79.3 (58.2-91.4) | 77.2 (56.8-89.7) |
| Kono | IVM | 71.5 (61.2-79.9) | 65.6 (54.2-75.5) |
|  | ALB | 70.7 (60.1-79.4) | 64.8 (53.3-74.8) |
| Moyamba | IVM | 62.7 (55.3-69.5) | 55.7 (47.4-63.6) |
|  | ALB | 62.8 (55.3-69.7) | 56.2 (47.2-64.8) |
| Port Loko | IVM | 57.8 (46.7-68.1) | 48.0 (39.6-56.5) |
|  | ALB | 57.2 (45.9-67.8) | 47.4 (39.1-55.9) |
| Pujehun | IVM | 64.4 (52.9-74.5) | 59.0 (47.1-70.0) |
|  | ALB | 63.9 (52.4-73.9) | 59.0 (47.1-70.0) |
| Tonkolili | IVM | 56.9 (48.3-65.1) | 50.1 (37.7-62.4) |
|  | ALB | 61.1 (45.3-74.8) | 59.6 (44.2-73.4) |
| RWA | IVM | 29.6 (10.8-59.4) | 24.0 (6.9-57.6) |
|  | ALB | 28.0 (8.5-62.1) | 28.0 (8.3-62.6) |
